# Improved production of tannase by *Klebsiella pneumoniae* using Indian gooseberry leaves under submerged fermentation using Taguchi approach

**DOI:** 10.1186/s13568-016-0217-9

**Published:** 2016-07-13

**Authors:** Mukesh Kumar, Amrinder Singh, Vikas Beniwal, Raj Kumar Salar

**Affiliations:** Department of Biotechnology, Chaudhary Devi Lal University, Sirsa, 125055 Haryana India; Department of Biotechnology Engineering, Ambala College of Engineering and Applied Research, Devsthali, Ambala, 133101 Haryana India; University Institute of Engineering and Technology, Kurukshetra University, Kurukshetra, 136119 Haryana India; Department of Biotechnology, Maharishi Markandeshwar University, Mullana, Ambala, 133203 Haryana India

**Keywords:** Tannase, Response surface methodology, *Klebsiella pneumoniae*, Central composite design, Taguchi orthogonal array, Agro-residues

## Abstract

Tannase (tannin acyl hydrolase E.C 3.1.1.20) is an inducible, largely extracellular enzyme that causes the hydrolysis of ester and depside bonds present in various substrates. Large scale industrial application of this enzyme is very limited owing to its high production costs. In the present study, cost effective production of tannase by *Klebsiella pneumoniae* KP715242 was studied under submerged fermentation using different tannin rich agro-residues like Indian gooseberry leaves (*Phyllanthus emblica*), Black plum leaves (*Syzygium cumini*), Eucalyptus leaves (*Eucalyptus glogus*) and Babul leaves (*Acacia nilotica*). Among all agro-residues, Indian gooseberry leaves were found to be the best substrate for tannase production under submerged fermentation. Sequential optimization approach using Taguchi orthogonal array screening and response surface methodology was adopted to optimize the fermentation variables in order to enhance the enzyme production. Eleven medium components were screened primarily by Taguchi orthogonal array design to identify the most contributing factors towards the enzyme production. The four most significant contributing variables affecting tannase production were found to be pH (23.62 %), tannin extract (20.70 %), temperature (20.33 %) and incubation time (14.99 %). These factors were further optimized with central composite design using response surface methodology. Maximum tannase production was observed at 5.52 pH, 39.72 °C temperature, 91.82 h of incubation time and 2.17 % tannin content. The enzyme activity was enhanced by 1.26 fold under these optimized conditions. The present study emphasizes the use of agro-residues as a potential substrate with an aim to lower down the input costs for tannase production so that the enzyme could be used proficiently for commercial purposes.

## Introduction

Tannase is an inducible enzyme that catalyzes the hydrolysis of ester bond (galloyl ester of an alcohol moiety) and the depside bond (galloyl ester of gallic acid) of hydrolysable tannins (Haslam and Stangroom 1996), releasing glucose, gallic acid and various galloyl esters of glucose. It is adaptive, intracellular/extracellular enzyme that belongs to esterase superfamily (Aguilar et al. 2007; Banerjee and Mahapatra 2012). Tannase has extensive applications in food, feed, beverage, brewing, pharmaceutical and chemical industries ranging from production of gallic acid, instant tea, coffee flavor refreshing drinks and acorn wine. Moreover, tannase is used in clarification of beer and fruit juices, improvement in the flavor of grape wine and manufacturing of animal feed (Das Mohapatra et al. 2009; Madeira et al. 2011; Belmares et al. 2004; Chavez-Gonzalez et al. 2012). Tannase is produced by different microbial sources like bacteria, yeast and fungi. Amongst these, most of the research work has focused on fungal organisms. However, use of fungal strain at industrial scale is limited due to its relatively slow growth rate and genetic complexity. Contrary to this, bacteria are characterized by a very high growth rate and they can be easily manipulated at genetic level. Bacteria also possess the ability to withstand extreme temperature and may be the potential source of thermostable tannase (Beniwal et al. 2015). Among bacteria, *Bacillus* and *Lactobacillus* genus have been widely investigated for the tannase production (Banerjee and Mondal 2001; Mondal et al. 2001a, b; Pinto et al. 2001; Murugan et al. 2007; Aguilar et al. 2007).

Microbial tannase is usually produced under submerged and solid state fermentation, each having certain advantages and disadvantages (Belmares et al. 2004). The main advantages of solid state cultures include simplicity, lower production costs, high enzyme yield and low wastewater. However, submerged cultures have advantages in process control, sterilization, whole substrate utilization, incubation time and ease of recovery of extracellular enzymes. In both of these fermentation techniques, high tannin containing materials are used as substrates. In spite of great industrial significance, a few of these applications have been commercially exploited due to the constraints imposed by the high cost of tannic acid, which acts as substrate for tannase production. In this regard, crude tannin obtained from a number of agro-residues could be used as a tannin rich natural substrate for cost effective tannase production. A number of natural substrates like jamun leaves, amla leaves (Kumar et al. 2007; Selwal et al. 2011), tamarind seed powder, baggase, ground nut oil cake, wheat bran and rice bran (Natarajan and Rajendran 2012), coffee pulp, tea residue (Sharma et al. 2014; Bhoite and Murthy 2015) have been used as substrates for tannase production under SSF. However, not much literature has been reported on the high level production and process economy for extracellular tannase from bacterial source under submerged fermentation conditions. Furthermore, reports on the optimization of tannase production are scarce. Optimization of fermentation parameters has been used to enhance the enzyme yield. Since, large numbers of variables are involved in the fermentation, different statistical methods are being used for the optimization of these parameters (Aravindan and Viruthagiri 2009; Natarajan and Rajendran 2012). Taguchi orthogonal array (OA) design is one of the statistical designs used to screen the most significant factors among the large number of independent variables. There are different kinds of designs available for optimization of significant fermentation factors, out of which, central composite design (CCD) is one of the most important experimental design being used in the optimization process (Montogomery 2000).

In the present study, an effort has been made to achieve cost effective production of tannase by *Klebsiella pneumoniae* KP715242 using *Phyllanthus emblica* leaves as a tannin source under submerged fermentation.

## Materials and methods

### Chemicals

All the chemicals used in the present investigation were of analytical grade and were procured from Himedia Biosciences.

### Microorganism and inoculum preparation

A tannase producing bacterium used in the present study was isolated from rhizospheric soil of *Acacia* species, identified as *K. pneumoniae* (GenBank Accession Number KP715242) on the basis of morphological, biochemical and16S ribosomal RNA gene sequence (Kumar et al. 2015).

Inoculum was prepared by growing a loopful of bacterium in a 250 ml Erlenmeyer flask containing 50 ml of basal medium (K_2_HPO_4_:0.5 g/l, KH_2_PO_4_:0.5 g/l, MgSO_4_:2.0 g/l, CaCl_2_: 1.0 g/l and NH_4_Cl: 3.0 g/l supplemented with 1 % tannic acid, pH 5.2) at 37 °C for 20 h.

### Substrates

A number of different agro-residues like leaves of Indian gooseberry (*Phyllanthus emblica*), Black plum (*Syzygium cumini*), Eucalyptus (*Eucalyptus glogus*) and Babul (*Acacia nilotica*) were collected from the local farms of Ambala Cantt, India. All the samples were collected aseptically in sample bags. These leaves were first dried at 60 °C in an oven and then finely pulverized to powdered form in a grinder mixer. The powder was stored in a dry place in sterilized bottles at room temperature and used as source of crude tannins in submerged fermentation.

### Estimation of tannin content

The tannin content in the crude extract of natural tannin substrates was determined by following the method of Hagerman and Butler (1978). Dried leaf powder was dissolved in distilled water and kept overnight at room temperature. After soaking, the mixture was boiled for 10 min and filtered. The filtered extract was used as source of crude natural tannin. One ml of extract was taken in a tube and 3 ml of BSA solution was added and kept for 15 min at room temperature. The tubes were centrifuged at 5000×*g* for 10 min, supernatant was discarded and pellet was dissolved in 3 ml of SDS-triethanolamine solution. One ml of FeCl_3_ solution was added and tubes were kept for 30 min at room temperature for color stabilization. Color was read at 530 nm against the blank.

### Mode of fermentation

Tannase production by *K. pneumoniae* KP715242 was carried out through submerged fermentation of crude tannin from different agro-residues at 35 °C at rotary shaker (100 rpm). Fermentation was carried out in 250 ml flask containing 50 ml of minimal medium containing K_2_HPO_4_: 0.5 g/l, KH_2_PO_4_: 0.5 g/l, MgSO_4_: 2.0 g/l, CaCl_2_: 1.0 g/l and NH_4_Cl: 3.0 g/l supplemented with 2 % crude tannin for 96 h. The medium was sterilized and the pH was adjusted to 5.2. Two percent of overnight grown culture was used as an inoculum. The biomass was separated by centrifugation and supernatant was used for tannase assay.

### Tannase assay

Enzyme solution (0.2 ml) was incubated with 0.3 ml of 1.0 % (w/v) tannic acid in 0.2 M acetate buffer (pH 5.5) at 40 °C for 40 min and then the reaction was terminated by the addition of 3 ml BSA (1 mg/ml), which precipitates the remaining tannic acid. A control reaction was also processed with heat denatured enzyme. The tubes were then centrifuged (7000×*g*, 10 min) and the precipitates were dissolved in 3 ml of SDS–triethanolamine (1 % w/v) solution. 1.0 ml of FeCl_3_ reagent (0.13 M) was added and kept for 15 min for stabilization of the color. The absorbance of both the test and control tubes was measured at 530 nm against the blank (without tannic acid). One unit of the tannase was defined as the amount of enzyme, which is able to hydrolyze 1 mM of substrate tannic acid in 1 min under assay conditions (Mondal 2001a, b).

### Statistical analysis

#### Taguchi orthogonal array (OA) design of experiment

Production of the enzyme in the fermentation process is influenced by a number of physical and nutritional variables. Tannase production by *K. pneumoniae* KP715242 using agro-residue (*Phyllanthus emblica* leaves) under submerged fermentation was optimized by evaluating the contribution of different process variables through Taguchi orthogonal array (OA) design of experiment (DOE). This statistical approach is used for the screening of most influential factors in the enzyme production. In the present report, eleven variables were considered and each was studied at two levels (−1 and +1). The minimum and maximum levels of each independent variable with their assigned levels are presented in Table 1.

**Table 1:** Selected culture condition in Taguchi OA design and assigned levels for tannase production from *Klebsiella pneumoniae* using agro-residue as substrate

S. no.	Factor	Level 1 (−1)	Level 2 (+1)
1	pH	4	6
2	Temperature	30	40
3	Incubation time (hrs)	48	96
4	rpm	50	150
5	Inoculum level (%)	1	5
6	Tannin extract (%)	1	3
7	Glucose (%)	0	2
8	NH_4_Cl (%)	0.1	0.5
9	KH_2_PO_4_ (%)	0.1	0.3
10	K_2_HPO_4_ (%)	0.1	0.3
11	MgSO_4_ (%)	0.05	0.1

An OA layout of L12 (2 × 11) generating 12 experiments was constructed at two coded levels for the proposed experimental design. The culture conditions studied were pH, temperature, incubation time, rpm, inoculum level, tannin extract, glucose, NH_4_Cl, KH_2_PO_4_, K_2_HPO_4_ and MgSO_4_.

### Optimization of most influential factors for maximum tannase production using response surface methodology (RSM)

The tannase production by *K. pneumoniae* KP715242 using agro-residues as tannin source was maximized by optimizing the four most contributing factors (as determined by using Taguchi orthogonal array (OA) design of experiment) through Response surface methodology (RSM). In order to examine the cumulative effect of four different culture conditions (independent variables) on tannase production, a central composite design (CCD) having 5 centre points leading to a total of 30 experiments was performed. The independent variables studied were pH (X1), incubation temperature (X2) (°C), incubation time (X3) (h) and tannin extract (X4) (%). The response (dependent variable) was tannase activity (U/ml). Each independent variable was studied at five coded levels. The minimum and maximum ranges of variables examined and the complete experimental plan with respect to their values in actual and coded form is listed in Table 2. The relation between the coded values and actual values were described as in the following Eq. 1:

1 $$x_{i} = (X_{i} - X_{0} )/\Delta X_{i} \quad {\text{I }} = { 1},{ 2},{ 3} \ldots ,{\text{ k}}$$

where xi is the coded value, Xi is the actual value of an independent variable; X_0_ is the real value of Xi at center point, ΔXi is the step change of the variable. The second-order model used to fit the response to the independent variables is shown in Eq. 2:

2 $$\begin{aligned}Y &= \beta_{0} + \sum\limits_{i = 1}^{k} {b_{i} } X_{i} + \sum\limits_{i = 1}^{k} {b_{ij} } X_{i}^{2} + \sum\limits_{{i_{i} < j}}^{k} {\sum\limits_{j}^{k} {b_{ij} } }\\ &\quad + X_{i} X_{j} + e \end{aligned}$$

where, i, j are linear, quadratic coefficients respectively, while ‘b’ is regression coefficient, Y is the tannase activity (U/ml), k the number of factors studied and optimized in the experiment, ‘e’ is random error and β_0_ is the intercept. A second-order regression analysis of the data was carried out to get empirical model that defines response in terms of the independent variables. Analysis of variance (ANOVA) was performed in coded level of variables to study the effects of independent variables. To determine the optimum level of selected variables for maximum tannase production, 3D graphs were generated to understand the effect of different factors individually and in combination with each other.

**Table 2:** Central composite design of the variables with tannase activity from *Klebsiella pneumoniae* as response using agro-residue as substrate

Run	pH	Incubation temp (°C)	Incubation time (h)	Tannin content (%)	Actual value	Predicted value
1	5.5{0}	37.5{0}	84{0}	0{−2}	0.0015	0.0013
2	4{−1}	25{−1}	120{1}	3{1}	0.0114	0.0113
3	5.5{0}	37.5{0}	84{0}	2{0}	0.0443	0.0416
4	7{1}	25{−1}	48{−1}	1{−1}	0.0148	0.0141
5	5.5{0}	12.5{−2}	84{0}	2{0}	0.0097	0.0123
6	5.5{0}	37.5{0}	84{0}	2{0}	0.0352	0.0416
7	5.5{0}	37.5{0}	84{0}	2{0}	0.0352	0.0416
8	5.5{0}	37.5{0}	84{0}	4{2}	0.0250	0.0243
9	4{−1}	50{1}	48{−1}	1{−1}	0.0156	0.0160
10	7{1}	50{1}	120{1}	3{1}	0.0136	0.0178
11	5.5{0}	37.5{0}	84{0}	2{0}	0.0444	0.0416
12	7{1}	50{1}	48{−1}	1{−1}	0.0151	0.0134
13	2.5{−2}	37.5{0}	84{0}	2{0}	0.0125	0.0101
14	7{1}	50{1}	48{−1}	3{1}	0.0139	0.0118
15	7{1}	25{−1}	48{−1}	3{1}	0.0173	0.0171
16	4{−1}	25{−1}	48{−1}	1{−1}	0.0012	0.0013
17	4{−1}	50{1}	120{1}	1{−1}	0.0211	0.0215
18	4{−1}	25{−1}	48{−1}	3{1}	0.0182	0.0118
19	5.5{0}	37.5{0}	84{0}	2{0}	0.0443	0.0416
20	5.5{0}	37.5{0}	84{0}	2{0}	0.0463	0.0416
21	5.5{0}	62.5{2}	84{0}	2{0}	0.0188	0.0198
22	8.5{2}	37.5{0}	84{0}	2{0}	0.0143	0.0142
23	4{−1}	25{−1}	120{1}	1{−1}	0.0074	0.0087
24	5.5{0}	37.5{0}	12{−2}	2{0}	0.0112	0.0119
25	7.5{1}	25{−1}	120{1}	3{1}	0.0241	0.0249
26	7.5{1}	25{−1}	120{1}	1{−1}	0.0183	0.0229
27	4{−1}	50{1}	120{1}	3{1}	0.0266	0.0265
28	7.5{1}	50{1}	120{1}	1{−1}	0.0277	0.0283
29	4{−1}	50{1}	48{−1}	3{1}	0.0221	0.0219
30	5.5{0}	37.5{0}	156{2}	2{0}	0.0254	0.0252

## Results

### Substrate

**Fig. 1:**
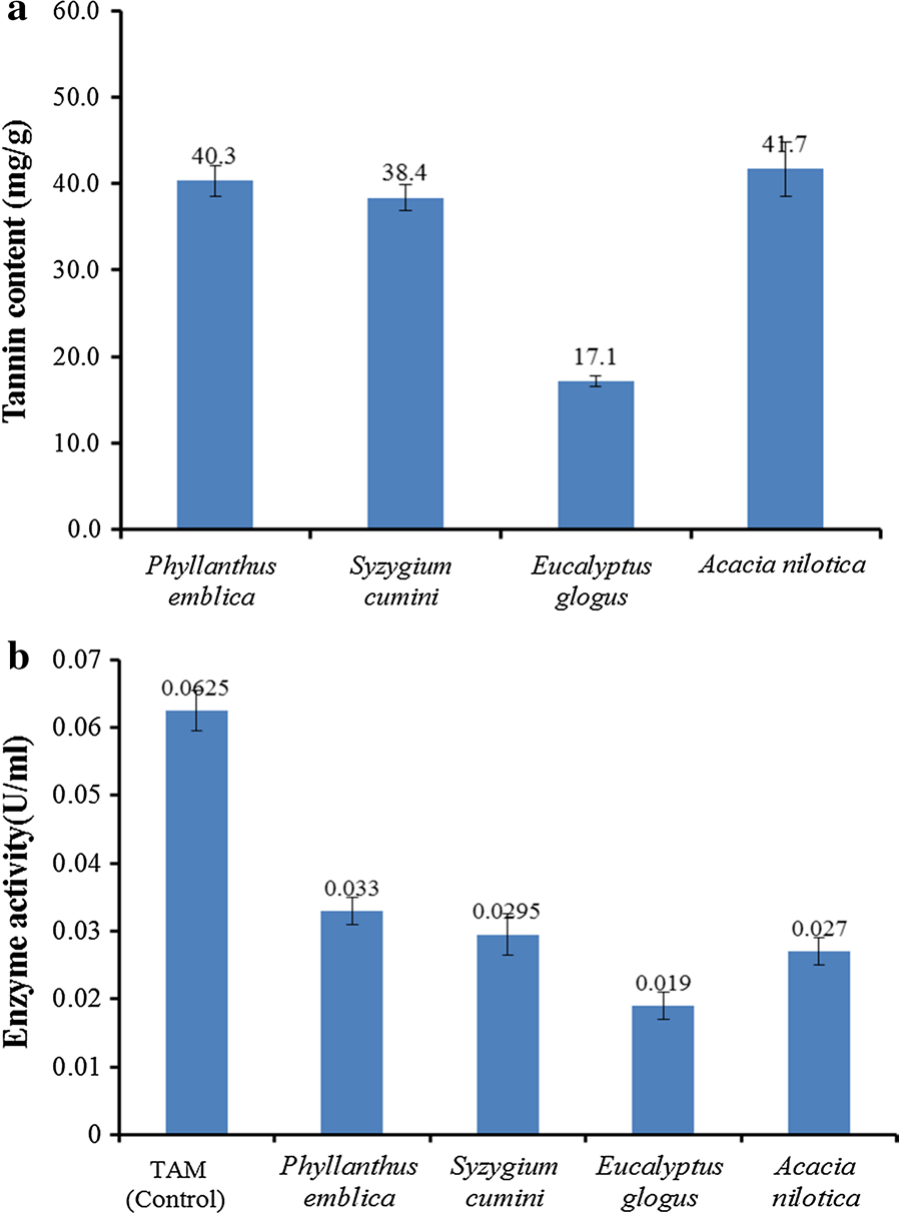
**a** Tannin content of different agro-residues used as substrate for tannase production, **b** production of tannase using different agro-residues as substrate

Tannin content of each substrate was estimated using the colorimetric method of Hagerman and Butler (1978). Figure 1a shows that maximum tannin content was present in *Acacia nilotica* leaves (41.6 mg/g dry leaves), followed by *Phyllanthus emblica* leaves (40.3 mg/g dry leaves), and *Syzygium cumini* (38.37 mg/g dry leaves). Lowest tannin content was observed in *Eucalyptus glogus* (17.1 mg/g dry leaves). All of these four substrates were used as sole tannin source for production of tannase under submerged fermentation. Maximum tannase production was observed in case of *Phyllanthus emblica* leaves (0.033 U/ml) suggesting it to be the best substrate out of the four agro-residues. This was followed by *Syzygium cumini* (0.029 U/ml), *Acacia nilotica* (0.027 U/ml) and *Eucalyptus glogus* (0.019 U/ml) leaves. However, the production was less in comparison to the pure tannic acid medium (TAM) used as control (Fig. 1b).

### Statistical analysis

#### Screening of most contributing fermentation factors through Taguchi orthogonal array (OA) design of experiment

Taguchi’s orthogonal arrays are highly fractional orthogonal designs. The Taguchi method is best used when there are an intermediate number of variables (3–50), few interactions between variables, and when only a few variables contribute significantly. In this design, OAs organizes the affecting variables and their levels in the way, most likely to affect the process. In contrast to factorial design, which involves testing of all the possible combinations, Taguchi employs a minimal number of trials by testing pairs of combinations and thus saves both time and resources.

In the present study, eleven factors were studied in 12 experiments for the screening of most influential factors affecting tannase production. The minimum and maximum ranges of variables studied and the full experimental plan with respect to their values in actual form is listed in Table 3. The influence or contribution of each factor at the assigned levels on tannase production by *K. pneumoniae* is presented in Table 4. It was observed that pH contributed maximally (23.62 %) towards tannase production followed by tannin extract (20.70 %), temperature (20.33 %) incubation time (14.99 %) and inoculum level (12.24 %). KH_2_PO_4_ was found to have least impact on overall production of tannase under the selected fermentation conditions (0.083 %).

**Table 3:** Taguchi OA design experiments for the selection of most contributing factors for tannase activity from *Klebsiella pneumoniae* using agro-residue as substrate

Run	pH	Temp	Incubation time (h)	Rpm	Inoculum level (%)	Tannin extract (%)	Glucose (%)	NH_4_Cl (%)	KH_2_PO_4_ (%)	K_2_HPO_4_ (%)	MgSO_4_ (%)	Response
1	4	30	96	150	5	1	0	0.1	0.3	0.3	0.1	0.0315
2	4	40	96	50	5	3	0	0.5	0.1	0.3	0.05	0.0330
3	6	30	48	150	5	3	0	0.5	0.3	0.1	0.05	0.0290
4	4	30	48	50	1	3	2	0.5	0.3	0.3	0.1	0.0298
5	6	30	96	50	5	3	2	0.1	0.1	0.1	0.1	0.0303
6	6	40	48	50	5	1	2	0.1	0.3	0.3	0.05	0.0295
7	6	40	96	50	1	1	0	0.5	0.3	0.1	0.1	0.0292
8	4	40	48	150	5	1	2	0.5	0.1	0.1	0.1	0.0305
9	4	40	96	150	1	3	2	0.1	0.3	0.1	0.05	0.0320
10	6	30	96	150	1	1	2	0.5	0.1	0.3	0.05	0.0281
11	4	30	48	50	1	1	0	0.1	0.1	0.1	0.05	0.0281
12	6	40	48	150	1	3	0	0.1	0.1	0.3	0.1	0.0305

**Table 4:** Contribution of selected factors on tannase production from *Klebsiella pneumoniae* using agro-residue as substrate

S. no.	Factor	% contribution
1	pH	23.620
2	Temperature	20.332
3	Incubation time	14.999
4	rpm	0.957
5	Inoculum level	12.244
6	Tannin extract	20.730
7	Glucose	0.374
8	NH_4_Cl	1.658
9	KH_2_PO_4_	0.083
10	K_2_HPO_4_	3.732
11	MgSO_4_	1.272

ANOVA (analysis of variance) and regression coefficients were used to evaluate the competence and fitness of the model for the Taguchi orthogonal array (OA) design experiments (Tables 5, 6). The model for orthogonal array experiments was significant with an F value of 18.352 as shown by Fisher’s F test. Values of “Prob > F” less than 0.0500 indicate model terms are significant, along with a very low probability value (P model > F = 0.0029), which was significant at 95 % confidence interval. Coefficient of variation is a measure of the accuracy and reliability of the model. In the present study, a low value of coefficient of variation (CV = 1.53 %) advocates the better precision and consistency of the experiments carried out. Determination coefficient (R^2^) was used to check the goodness fit of the model. In the present design, value of the determination coefficient (R^2^ = 0.957) specifies that 95.7 % of the total variation in the tannase production is ascribed to the independent variables. The predicted R^2^ of 0.750 for tannase production is in reasonable agreement with the adjusted R^2^ of 0.904 i.e. the difference is less than 0.2. A comparison between range of predicted values at the design points and the average prediction error shows adequate precision. Adeq precision measures the signal to noise ratio. A ratio greater than 4 is desirable. In this case ratio is 14.89 indicates an adequate signal. The model was found to be significant for production within the range of variables employed.

**Table 5:** ANOVA (analysis of variance) of factorial Taguchi OA design for the factors contributing towards tannase production from *Klebsiella pneumoniae* using agro-residue as substrate

Source	Sum of squares	Df	Mean square	F value	P value Prob > F	
Model	2.36577E−05	6	3.94295E−06	18.35201	0.00290762	Significant
pH	5.8417E−06	1	5.8417E−06	27.18955	0.003425898	
Temperature	5.02849E−06	1	5.02849E−06	23.40452	0.004723958	
Incubation time	3.70963E−06	1	3.70963E−06	17.26607	0.008864846	
Inoculum level	3.02807E−06	1	3.02807E−06	14.09379	0.013236736	
Tannin extract	5.12684E−06	1	5.12684E−06	23.86229	0.004533592	
K_2_HPO_4_	9.22965E−07	1	9.22965E−07	4.295839	0.092927125	
Residual	1.07426E−06	5	2.14851E−07			
Cor total	2.47319E−05	11

**Table 6:** Statistical analysis for selection of contributing factors towards tannase production from *Klebsiella pneumoniae* using agro-residue as substrate

Std. dev.	0.0005	R squared	0.957
Mean	0.0301	Adj R squared	0.904
C.V. %	1.5393	Pred R squared	0.750
PRESS	0.0000	Adeq precision	14.895

#### Optimization of most contributing factors for maximum tannase production through response surface methodology

The effect of four most influential factors (pH, temperature, incubation time, and tannin extract) on tannase activity from *K. pneumoniae* KP715242 using *P. emblica* leaves as substrate under submerged fermentation was studied and optimized with Central composite design (CCD) of Response surface methodology (RSM). In this technique, each independent variable was studied at five coded levels and thirty experimental runs were performed as designed by central composite design to optimize the four most contributing parameters. The experimental and predicted results of tannase yield are given in Table 2. It was observed that the predicted values for tannase production are in good agreement with observed values.

### Model validation

The significance of the quadratic regression model was evaluated by Fisher’s test (F test) and ANOVA (Table 7). The F value corresponding to tannase was 13.21 with a very low probability value which justified the significance of the model. The R^2^ was significant at the level of 92.4 % in tannase production which inferences that only 7.6 % of total independent variable was not explained by the model. It indicates that all the independent factors contribute to a combined effect to maximize the production of tannase. The predicted R^2^ of 0.690 for tannase production is in reasonable agreement with the adjusted R^2^ of 0.854. The adequate precision measures the signal to noise ratio in the model. This ratio greater than 4 is desirable. In this study, the ratio of 12.69 indicates an adequate signal for the model (Table 8). The insignificant lack of fit value also indicated model can be used to navigate the design space. The model was found to be significant for production within the range of variables employed. The final predictive equation was as follows:

**Table 7:** ANOVA (Analysis of variance) for response surface quadratic model for optimization of tannase production of *Klebsiella pneumoniae* using agro-residue as substrate

Source	Sum of squares	Df	Mean square	F value	P value Prob > F	
Model	0.0040867	14	0.0002919	13.2114	5.553721E−06	Significant
A-pH	0.0000257	1	0.0000257	1.1619	2.981019E−01	
B-Incubation temp.	0.0001554	1	0.0001554	7.0332	1.811788E−02	
C-Incubation time	0.0001503	1	0.0001503	6.8041	1.976303E−02	
D-tannin content	0.0002212	1	0.0002212	10.0123	6.416836E−03	
AB	0.0001657	1	0.0001657	7.4990	1.523655E−02	
AC	0.0000110	1	0.0000110	0.5001	4.903135E−01	
AD	0.0001000	1	0.0001000	4.5249	5.040503E−02	
BC	0.0000095	1	0.0000095	0.4321	5.209274E−01	
BD	0.0000658	1	0.0000658	2.9770	1.049926E−01	
CD	0.0000352	1	0.0000352	1.5945	2.259671E−01	
A^2^	0.0012408	1	0.0012408	56.1576	1.907611E−06	
B^2^	0.0011574	1	0.0011574	52.3822	2.889849E−06	
C^2^	0.0008288	1	0.0008288	37.5096	1.944786E−05	
D^2^	0.0012493	1	0.0012493	56.5403	1.831271E−06	
Residual	0.0003314	15	0.0000221			
Lack of fit	0.0002059	10	0.0000206	0.8205	6.318597E−01	Not significant
Pure error	0.0001255	5	0.0000251			
Cor total	0.0044181	29

**Table 8:** Statistical analysis for tannase production from *Klebsiella pneumoniae* using agro-residues as substrate

Std. dev.	0.00470055	R squared	0.92498466
Mean	0.02121422	Adj R squared	0.85497035
C.V. %	22.1575512	Pred R squared	0.69062148
PRESS	0.00136688	Adeq precision	12.6996545

Tannase activity (Y) = 0.041588233 + 0.001034255 *A + 0.002544608 * B + 0.002502813 * C + 0.003036066 * D−0.003218036 * AB + 0.000831001 * AC − 0.00249973 * AD + 0.000772455 * BC − 0.002027572 * BD − 0.001483871 * CD − 0.006725918 * A^2^ − 0.006495897 * B^2^ − 0.005496905 * C ^2^ − 0.006748795  * D^2^ where, Y represents the tannase produced as a function of the coded levels of pH (A), incubation temperature (B), incubation time (C) and tannin extract (D).

### Three dimensional response surface plots

**Fig. 2:**
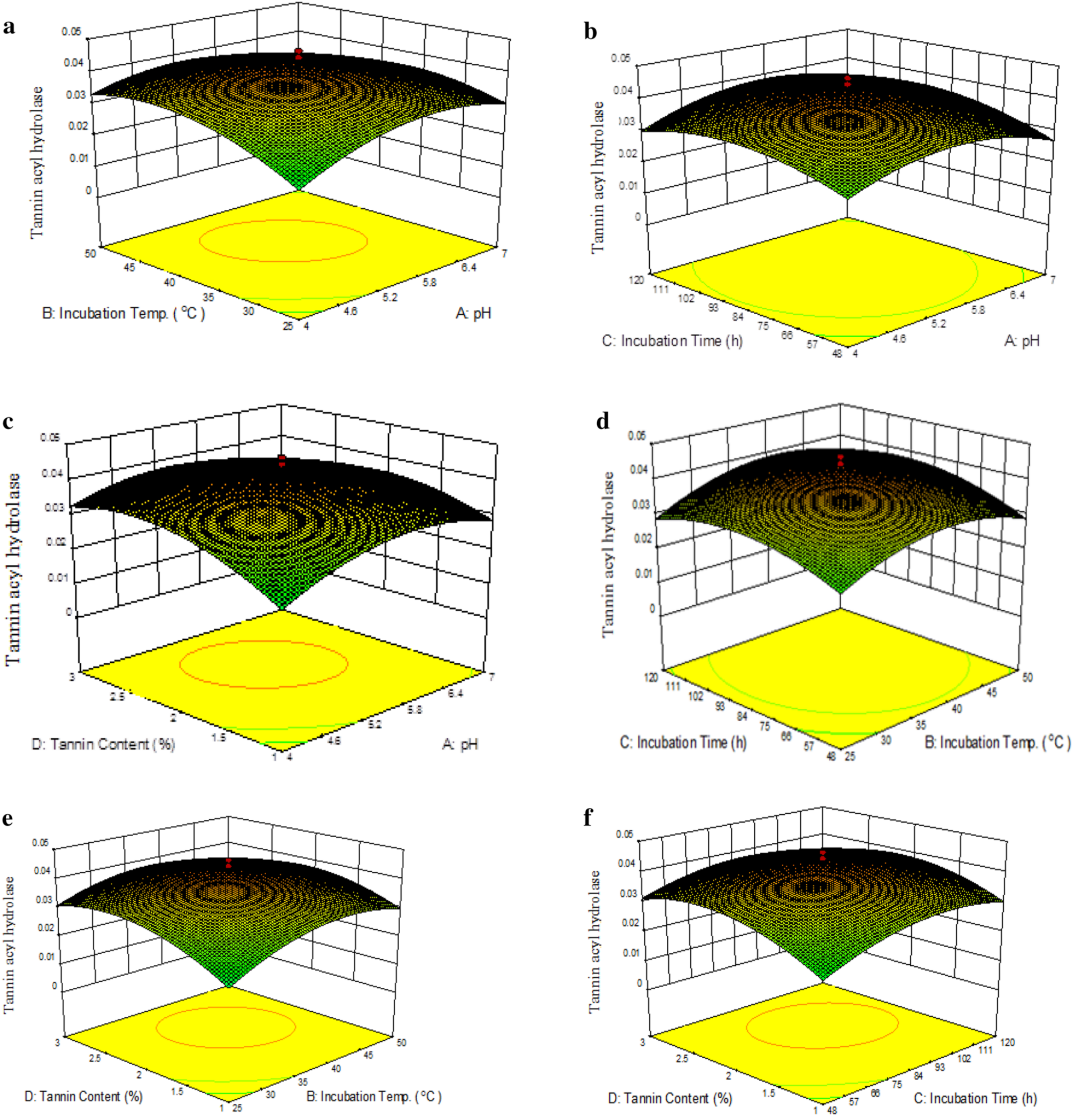
**a** Effect of incubation temp and pH on tannase production keeping incubation time and tannin content at zero level (coded), **b** effect of incubation time and pH on the production of tannase while incubation temp and tannin content were held at zero level (coded), **c** effect of tannin content and pH on the production of tannase. Other variables incubation temp and incubation time were kept at zero level (coded), **d** effect of incubation time and temperature on the production of tannase keeping pH and tannin content at zero level (coded), **e** effect of tannin content and incubation temp on the production of tannase. Other variables pH and incubation time were held at zero level (coded) and **f** effect of tannin content and incubation time on the production of tannase. Other variables pH and incubation temp were held at zero level (coded)

The three-dimensional (3D) response surfaces plots (Fig. 2a–f) were designed on the basis of the model equation to investigate the interaction among the above factors as well as to attain the optimum level of each factor for maximum production of tannase using *K. pneumoniae* KP715242. In these 3D response surface plots, two factors were studied within their predefined range while keeping the other two factors at their optimum level. On the basis of these three dimensional response surfaces plots it is revealed that increase in pH and incubation temperature leads to maximum tannase production at the optimum values of 5.52 and 39.72 °C, respectively. The enzyme production declined with further increase in these factors. The maximum tannase production was obtained when the fermentation was carried out for 91.82 h with a tannin content of 2.17 %. A further increase in the incubation time and tannin content beyond the optimum level resulted in decreased enzyme production.

## Discussion

The present study was carried out for production of low cost tannase by *K. pneumoniae* KP715242 by using agro-residues as natural substrate. The tannase production was further improved through Taguchi methodology based optimization. The choice of the substrate for tannase production is largely dependent upon its cost and availability. In this study, different agro-residues were tested for tannase production under submerged fermentation and it was observed that the high tannase productivity in case of *P. emblica* and *S. cumini* leaves corresponded to their high tannin content. The present results are in close accordance to Kumar et al. (2007) who studied tannase production under solid state fermentation using different tannin rich substrates like ber leaves (*Z. mauritiana*), jamun leaves (*S. cumini*), amla leaves (*P. emblica*) and jawar leaves (*Sorghum vulgaris*). Jamun and Amla leaves were found to be the best substrate for enzyme production. Selwal et al. (2011) used different agro-residues like amla (*P. emblica*), ber (*Z. mauritiana*), jamun (*S. cumini*), Jamoa (*Eugenia cuspidate*) and keekar (*Acacia nilotica*) leaves as substrate for tannase production by *P. atramentosum* KM. Maximum extracellular tannase production was observed in *P. emblica* containing medium. Mohapatra et al. (2006) used eight different tannin containing substrates (*Acacia auriculiformis*, *Casuarina equisetifolia*, *Psidium guazava*, *Anacardium occidentale*, *Delonix regia*, *Eucalyptus tereticornis*, *Cassia fistula*, *Ficus benghalensis*) for the production of tannase through submerged fermentation by *Bacillus licheniformis* KBR6 and reported that the extract of *A*. *auriculiformis* proved to be the best substrate yielding maximum tannase production within 15–18 h of growth in all the extracts except *Eucalyptus tereticornis*. Varadharajan et al. (2015) investigated various agro-wastes as substrates for the tannase production by *Aspergillus oryzae* by submerged fermentation and found pomegranate rind extract as the best substrate with a tannase yield of 138.12 IU/ml.

Since the growth of microorganisms as well as enzyme production through microbial fermentation is dependent up on a number of physico-chemical parameters, it becomes imperative to optimize these culture conditions for maximum enzyme production. The statistical models are extensively used for the screening and optimization of different process conditions. In the present study, statistical designs namely Taguchi orthogonal array design and response surface methodology were used to screen the fermentation conditions and maximize the tannase production by *K. pneumoniae* KP715242 in submerged fermentation using *P. emblica* leaves as substrate.

The four most significant factors contributing maximally towards tannase production as revealed by Taguchi OA are pH (23.62 %) followed by tannin extract (20.70 %), temperature (20.33 %) incubation time (14.99 %). Every microorganism possesses a specific pH for stimulation and subsequent expression of a gene of the enzyme. Maximum bacterial tannase biosynthesis has been reported in acidic (pH 4.5) to neutral (pH 7.0) pH range. Tannase action results in the breakdown of tannic acid into gallic thereby leading to an acidic environment. Therefore, fermentation at lower and higher pH becomes unfavorable to enzyme production (Jana et al. 2013). The crude tannin extract provides tannic acid that in addition to being the carbon source also acts as a vital factor for bacterial growth, stimulation and expression of the tannase gene (Mondal and Pati 2000). Microbial biosynthetic pathways and the transport mechanism of various metabolites across the bacterial cell membrane are largely dependent on the temperature. Low temperature may lead to decreased tannase yield probably due to lower transport of substrate across the cell. Near the optimum temperature, the rate of reaction is increased possibly due to increase in the kinetic energy of reacting molecules. Enzyme production is inhibited at higher temperatures because of the denaturation of metabolic pathways (Jana et al. 2013). The low contribution of glucose may be due to the fact that its requirement is probably compensated by the endogenously glucose produced as a result of tannase action on the hydrolysable tannins present in the crude tannin. The crude tannin may also serve as a source of other salts resulting in the low contribution of these salts towards enzyme production.

In view of the cost effective production of bacterial tannase using agro-residues as substrate and its subsequent industrial importance, it is imperative to optimize the culture conditions for maximum enzyme production. The effect of four most contributing process parameters namely pH, temperature, tannin content and incubation time on tannase enzyme activity in submerged fermentation using *K. pneumoniae* KP715242 was studied and optimized with central composite design of RSM. Optimum conditions for maximum enzyme production were 5.52 pH, 39.72 °C temperature, 91.82 h of incubation time and 2.17 % tannin content. Under these optimum conditions, the bacterium yielded 0.0415 U/ml of tannase which is 1.26 fold higher than the 0.033 U/ml of tannase initially produced under un-optimized conditions.

Figure 2a–c illustrates the interaction of pH with incubation temperature, incubation time and tannin content respectively. Tannase activity was found to increase with increase in pH to optimum value of 5.52. As pH increased beyond 5.52, tannase activity declined suggesting adverse effect on tannase production. Changes in the pH may cause protonation or deprotonation of amino acids and active site of the enzyme thereby culminating in altered tannase activity. Further, conformational changes in tannase structure in response to amino acid ionization may also affect enzyme activity (Sabu et al. 2006). Acidic pH preferred the maximum activity whereas it decreased in the alkaline range (Raghuwanshi et al. 2011). Figure 2d, e depicts the interaction effects of fermentation temperature with incubation time and tannin content respectively. It was observed that tannase activity increased with increase in temperature with an optimum value of 39.72 °C. Further increase in temperature afar this value led decrease in tannase activity. Figure 2f reveals interaction of incubation time and tannin content. Tannase activity increased with the increase in incubation time and tannin content to optimum values of 91.82 h and 2.17 %, respectively. After the optimum period of fermentation, tannase activity declined possibly due to exhaustion of nutrients in the medium. The present results are in close accordance to Mohan et al. (2014a, b) who studied the applications of RSM for the production of tannase by *Aspergillus foetidus* (MTCC 3557) using redgram husk as substrate under submerged fermentation. The reported optimum conditions were tannin content of 3.1 %, fermentation period of 97 h, temperature of 35.5 °C and pH of 5.5. Similar kind of results have been reported in case of tannase production from *Aspergillus awamori* MTCC 9299 and *Aspergillus flavus* (Beniwal and Chhokar 2010; Mohan et al. 2014a, b). Bhoite et al. (2015) also studied production of tannase by *P. verrucosum* using coffee pulp as substrate and obtained maximum tannase production at 96 h of fermentation period.

## Conclusions

Large scale industrial application of tannin is very limited owing to its high production costs. In the recent past, several studies showing different strategies to lower down the production cost were largely focused on the screening or development of tannase producing microbial strains. The present study reports the cost effective production and statistical optimization of tannase through biodegradation of agro-residues like *P. emblica* leaves by *K. pneumoniae* KP715242. The results obtained in this investigation indicate that *P. emblica* leaves hold the potential to be a candidate substrate for production of tannase in an economical and environmental friendly manner. However, the tannin levels in different plants vary with agro-climatic conditions, therefore, further studies are required to screen industrial waste produced from various plants for commercial utilization of the waste.

## Abbreviations

CCD: central composite design
RSM: response surface methodology
BSA: bovine serum albumin
SDS: sodium dodecyl sulfate
OA: Taguchi orthogonal array
DOE: design of experiment
ANOVA: analysis of variance
